# NF-κB and Pancreatic Cancer; Chapter and Verse

**DOI:** 10.3390/cancers13184510

**Published:** 2021-09-07

**Authors:** John Silke, Lorraine Ann O’Reilly

**Affiliations:** 1 Inflammation Division, Walter and Eliza Hall Institute of Medical Research (WEHI), Parkville, VIC 3052, Australia; silke@wehi.edu.au; 2 Department of Medical Biology, University of Melbourne, Parkville, VIC 3010, Australia

**Keywords:** pancreatic cancer, PDAC, NF-κB, pancreatitis, inflammation, tumor microenvironment, therapy, Smac mimetics

## Abstract

**Simple Summary:**

The incidence of pancreatic cancer is increasing but there has been little progress in the diagnosis and survival rates of this lethal cancer for decades. Understanding the biological mechanisms and defining how pancreatic cancer develops from normal pancreas tissue, to early lesions and then tumor is vital in developing better treatment options. Unrelenting inflammation in the pancreas significantly increases the risk of developing precursor lesions which result in pancreatic cancer. This inflammatory environment can result in the “switching on” of signaling pathways in the pancreas that influences many factors such as cell survival and turnover involved in tumor initiation, development and spread. In this review we discuss in detail how components of one such pathway, the NF-κB signaling network, are involved at various stages of pancreatic cancer development and in the cellular milieu of this cancer. We also discuss how this signaling pathway could be potentially “switched off” or regulated using new inhibitors and how these agents may be coupled with conventional and/or other therapies for better patient treatment outcomes.

**Abstract:**

Pancreatic Ductal Adenocarcinoma (PDAC) is one of the world’s most lethal cancers. An increase in occurrence, coupled with, presently limited treatment options, necessitates the pursuit of new therapeutic approaches. Many human cancers, including PDAC are initiated by unresolved inflammation. The transcription factor NF-κB coordinates many signals that drive cellular activation and proliferation during immunity but also those involved in inflammation and autophagy which may instigate tumorigenesis. It is not surprising therefore, that activation of canonical and non-canonical NF-κB pathways is increasingly recognized as an important driver of pancreatic injury, progression to tumorigenesis and drug resistance. Paradoxically, NF-κB dysregulation has also been shown to inhibit pancreatic inflammation and pancreatic cancer, depending on the context. A pro-oncogenic or pro-suppressive role for individual components of the NF-κB pathway appears to be cell type, microenvironment and even stage dependent. This review provides an outline of NF-κB signaling, focusing on the role of the various NF-κB family members in the evolving inflammatory PDAC microenvironment. Finally, we discuss pharmacological control of NF-κB to curb inflammation, focussing on novel anti-cancer agents which reinstate the process of cancer cell death, the Smac mimetics and their pre-clinical and early clinical trials.

## 1. Introduction

Pancreatic cancers are associated with a poor prognosis and are one of the leading causes of cancer mortality worldwide [1,2,3]. They are predicted to become the second most common cause of cancer death in the developed Western world by 2030 [4,5]. The disease often presents only when the tumor has already metastasized and this late diagnosis is a leading factor influencing the poor disease outcome [6]. Curative surgical options remain limited to those rare patients with localized tumor [7]. Notwithstanding the recent advances in surgical interventions [8] and treatment modalities, there has been almost no improvement in the current 5-year survival rate of 9% [5,6,7,9]. Cancers of the pancreas are mainly divided into two types; stromal-rich pancreatic ductal adenocarcinomas (PDAC), arising from the pancreatic exocrine tissue (predominantly ductal cells) and pancreatic neuroendocrine tumors emanating from pancreatic endocrine tissue (PanNET, less than 5% of pancreatic cancers) [10]. This review is restricted to a discussion of PDAC because it is the most prevalent and aggressive form of pancreatic cancer.

There are strong non-modifiable familial and specific syndrome risk factors (e.g., Lynch syndrome [2,10]) for PDAC, that are associated with certain genetic variations or mutations [6,7,11]. However, these only account for 5–10% of new cases [12,13]. The majority of PDAC evolves spontaneously from microscopic lesions of the pancreas and there are a number of risk factors that can accelerate this process. These include smoking, alcohol, obesity, dietary factors [6] and infection with *H. pylori* [14]. Inflammation of the pancreas (pancreatitis) that we hypothesize may smoulder for decades, with an accompanying elevated cytokine/chemokine milieu, may instigate genetic mutation and PDAC development.

PDAC is generally believed to develop through a step-wise series of genetic mutations from normal mucosa to precursor lesions and then invasive malignant disease [15,16]. However, recent evidence also suggests an important role for “catastrophic” chromosomal deletion and rearrangement events called chromothripsis in the initiation of PDAC [10]. Pancreatic Intraepithelial Neoplasia (PanIN) is the most common precursor lesion, originating from the small microscopic ducts as a non-invasive microscopic lesion. PanINs are graded to chart progressive morphological severity [16,17,18] and it has been postulated that PanIN could have a role in pancreatitis development and that the accompanying epithelial trauma and repair cycles may fuel further neoplastic development [16]. Pre-malignant low grade PanIN lesions are common in older populations (>75% of post-mortem studies [19]), however, it is predicted by mathematical modelling of genetic studies to take about 12 years to progress from high grade PanIN to PDAC, with the overall chance of progression about 1% [20]. Nevertheless, supplementary risk factors, such as first-degree relatives with PDAC and/or chronic pancreatitis, greatly increase the likelihood that PanIN1 lesions will progress to PDAC [12,21].

The progression of PanINs mirrors the progressive accumulation of molecular irregularities [22], from mutational activation of *KRAS* [23,24], inactivation of *INK4A*/*ARF* [25] to higher grade PanINs/PDAC associated with additional driver mutations (*P16*, *CDNK27*, *TP53*, *SMAD4*, *CDKN2A*, *SMAD4*, *BRCA1*, and *BRCA2*) [15,26,27]. Genomic analysis has permitted stratification of PDAC into four sub-groups (pancreatic progenitor, squamous, immunogenic and aberrantly differentiated exocrine) [28,29,30], identifying this tumor type as having a complex heterogeneous molecular and microenvironmental landscape.

Recent modifications of chemotherapy combination treatment regimens, such as FOLFIRINOX [31] have modestly increased 5 year survival rates [31,32,33,34,35]. Immunotherapies such as immune checkpoint blockade or engineered (CAR) T cells have been efficacious in certain sub-groups of cancer patients but to date have been disappointing for PDAC [36,37,38]. This may be due to the fact that PDAC is a highly aggressive malignancy, characterized by an immunosuppressive microenvironment of dense fibrotic stroma, poor T cell infiltration, variable mutational burden and possible chromothripsis.

Most human solid cancers are initiated by constant inflammation over extended periods of time [39,40,41,42] and this presumably is the case for PDAC [6,43]. Numerous studies [2] have reported an increased risk for PDAC associated with both Type 1 [44] and Type 2 diabetes [45], with PDAC often manifesting in the early stages, appearing as new onset diabetes [2,46]. Chronic inflammation and cancer development are now well accepted to be linked through the NF-κB activation cascade [39,40,41,42,47,48,49] with NF-κB constitutively activated in many cancers including PDAC [50,51,52,53]. Both the canonical and non-canonical arms of the NF-κB pathway are known to play important roles in PDAC development, progression and drug resistance [50,51,53].

The incidence of PDAC is increasing and, there are a lack of effective treatment options. Research into the early events in the pancreas precipitating PDAC development might allow “at risk” patients to be identified at an earlier stage in their disease with the possibility of intervening to prevent or slow progression. The generation of animal models that accurately recapitulate the progression and early biology of this cancer will allow greater insight into progression and also provide opportunities to test new therapeutic approaches. This review will therefore examine the role of NF-κB in pancreatic inflammation and PDAC development with a particular emphasis on the role of NF-κB in the cellular milieu in order to advance understanding of how this inflammation can be curbed to prevent PDAC. We also summarize how the roles of NF-κB in the tumor microenvironment; control of survival, invasion and metastasis could be tempered by novel NF-κB-based therapy approaches, with a particular focus on the Inhibitor of Apoptosis Proteins (cIAP1/2).

## 2. NF-κB, a Potent and Versatile Transducer of Inflammatory Signals

NF-κB is a family of inducible transcription factors expressed in all tissues and cell types, that becomes activated by a wide range of signals and is required in both innate and adaptive immunity to rapidly activate cellular responses [48,49,54,55,56]. The minimal active unit is usually a homo or hetero dimer of two NF-κB proteins and these dimers also have critical roles in normal cell homeostasis, proliferation, survival and differentiation [47,57,58,59,60]. Its function is mediated through the responses to a diverse range of stimuli and its ability to cause transcriptional activation or suppression of >150 target genes (accessed on 07 July 2021, http://www.bu.edu/nf-kb/generesources/target-genes; NF-κB target gene list, maintained by the Gilmore laboratory). Given the targets of NF-κB it is not hard to imagine how dysregulated NF-κB activation can contributes to the pathogenic processes of various inflammatory diseases [61]. In tumorigenesis for example, NF-κB regulates pro-inflammatory genes such as cytokines and chemokines (e.g., TNF, IL-1, IL-6, IL-8, IL-17), proliferative (cyclin D1, Cyclin E) and anti-apoptotic genes (Bcl-2, Bcl-xL), which can enhance tumor growth and survival [39,62]. NF-κB also regulates genes regulating stemness, epithelial-to-mesenchymal transition (EMT) and angiogenesis, promoting tumor growth, metastasis and therapy resistance [42,63]. While much is known about the NF-κB family of proteins, how this is translates in a cell-type specific and cellular context dependent manner, and how sustained NF-κB activation can lead to nascent tumorigenesis are still far from completely understood.

### 2.1. The NF-κB Family

**Figure 1:**
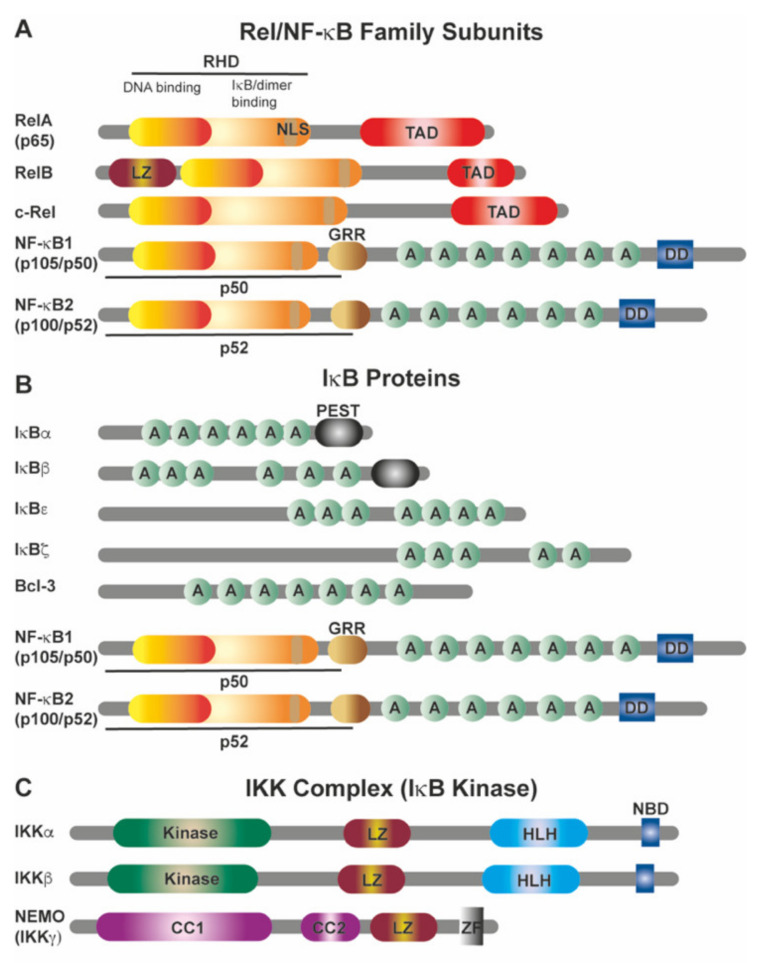
Structural Domain organisation of the Rel/NF-κB signalling pathway components. The mammalian NF-κB family members share a conserved Rel homology domain (RHD), nuclear localisation sequence (NLS) and a transcriptional activation domain (TAD) (**A**). The glycine rich region (GRR) in the p105 and p100 proteins signal for restricted proteasomal processing to generate the p50 and p52 proteins, respectively (black lines). (**B**) The transcriptional activity of NF-κB is regulated by the IκB proteins; IκBα, IκBβ, IκBɛ, IκBζ, BCL-3, IκBNS, p100, and p105. (**C**) The IKK complex consists of the catalytic kinase subunits IKKα (IKK1), IKKβ (IKK2) and the regulatory subunit NEMO (IKKγ). Both IKKα and IKKβ possess a helix-loop-region (HLH) and a leucine zipper domain (LZ), which mediate both homo- and hetero-dimerization of these proteins. IKKα and IKKβ interact with NEMO through their NEMO binding domain (NBD), which contains coiled coil domains (CC) and a leucine zipper (ZF). Protein domains typifying each protein family; (A) Ankyrin repeat domain; (DD) death domain; (GRR) glycine-rich region; (PEST) proline-rich, glutamic acid-rich, serine-rich, and threonine-rich.

NF-κB comprises two units from a family of five related, DNA binding, proteins; NF-κB1 (p50/p105), NF-κB2 (p100/p52), c-Rel, RelB and RelA (p65) [47,57] (Figure 1). The NF-κB1(p50)/RelA(p65) heterodimer is the most abundant form of NF-κB dimer and is found in almost all cell types [47]. p65/RelA, RelB, and c-Rel contain carboxy-terminal transactivation domains (TAD) and dimers containing these are usually held inactive in the cytoplasm by distinct, Ankyrin-Repeat containing, inhibitory, IκB proteins. However, in their unprocessed form, NF-κB1 (p105), NF-κB2 (p100) also contain Ankyrin-Repeats and can therefore act like IκB proteins and inhibit nuclear localization and the transcriptional activity of NF-κB dimers [47,64] (Figure 1). NF-κB1 tends to be constitutively processed and p50 is therefore readily detected in most cells, on the other hand p100 is usually processed to p52 only in response to an NF-κB activating stimulus and is therefore the predominant form seen in unstimulated cells.

**Figure 2:**
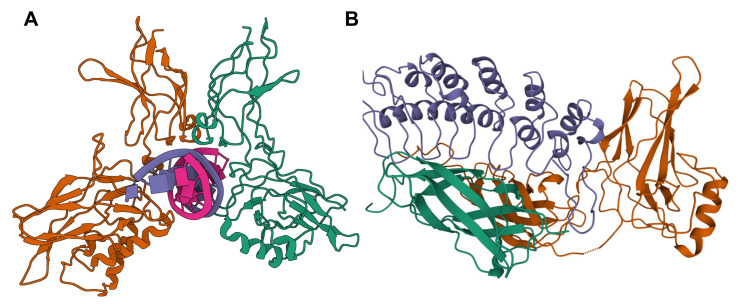
NF-κB/DNA complex structures. (**A**) Ribbon representation of the crystal structure of murine NF-κB1 (p50, residues 39–364, green) and RelA (p65, residues 19–291, orange) heterodimer bound to the kappaB DNA of the intronic enhancer of the immunoglobulin light-chain gene (blue/red) [71] generated by Mac PyMol from PDB ID 1VKX. (**B**) Ribbon representation of the crystal structure of human IκBα (residues 67–302, blue) bound to the murine NF-κB1 (p50, dimerisation domain residues 245–363, green) and murine RelA (p65, residues 19–304, orange heterodimer [72]) from PDB ID 1IKN.

Like the DNA binding units, the IκB proteins form a family of related proteins: IκBα, IκBβ, IκBɛ, IκBζ, BCL-3, IκBNS, p100, and p105. While they do not all exclusively restrict the DNA binding units to the cytoplasm and in some cases can facilitate transcription [65,66,67,68], those that do must be inactivated in order to allow the DNA binding dimers to enter the nucleus. This usually involves phosphorylation leading to degradation or proteolytic processing of the IκB subunit thereby liberating the DNA binding dimers to translocate to the nucleus [48,49]. The kinase complex that phosphorylates the IκB subunit is composed of IKK1 (IKKα), IKK2 (IKKβ) and the scaffold protein (NEMO (IKKγ)) [69]. Having pre-formed dimers poised to enter the nucleus allows the NF-κB transcriptional response to be very rapid. Furthermore, because NF-κB often promotes transcription of IκB family members this method of regulation also allows the response to be finely tuned and turned off once NF-κB has sufficiently activated transcription. The classic view is that NF-κB is released from IκB inhibition and translocates to the nucleus to induce transcriptional activation by binding to κB sites on target genes (Figure 2). However, in truth, NF-κB bound to IκBα constantly shuttles between the cytoplasm and nucleus with the dominant activity of IκBα maintaining cytoplasmic localization [70], thus also explaining how nuclear NF-κB can be exported out of the nucleus by freshly synthesized IκBs.

### 2.2. Downstream Signaling of Canonical and Non-Canonical NF-κB Activation

**Figure 3:**
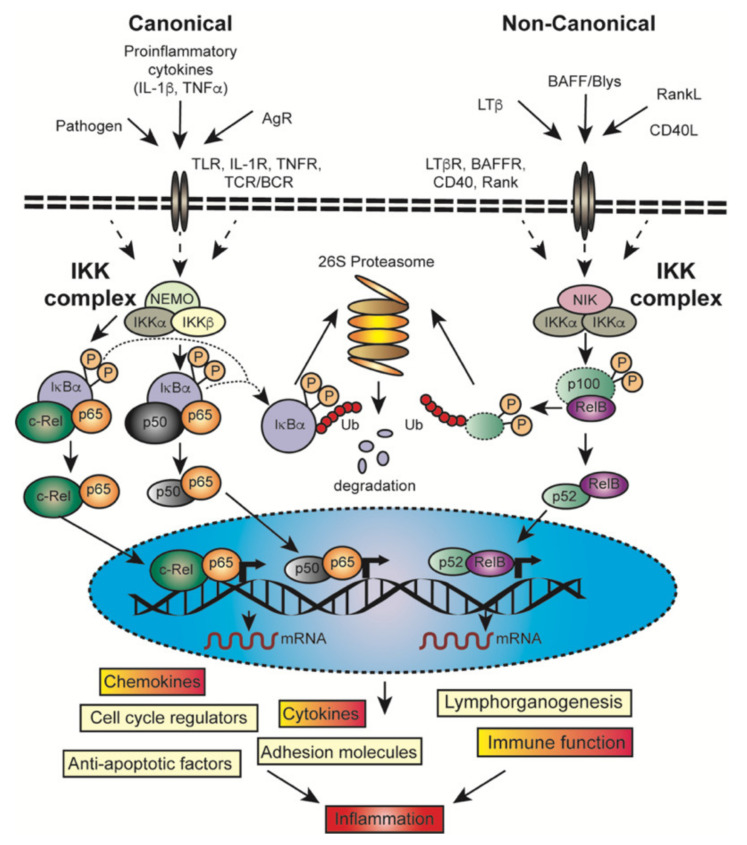
The canonical and non-canonical NF-κB signalling pathways. Simplified diagrams of NF-κB signaling, (left): Stimulation of TOLL-like receptors (TLRs), infection or ligand binding (e.g., TNF) to cell surface receptors (e.g., TNF-R1) trigger c-REL, NF-κB1 (p105/p50) or p65/RelA and activation of the inhibitory IKK (IκB kinase) complex. This complex is recruited to adaptor proteins (TRAFs or RIP kinases (dashed arrows)), activating the IKK complex, resulting in phosphorylation of Iκ proteins, priming them for ubiquitination and proteasome degradation. NF-κB dimers (NF-κB1 (p50) and c-Rel pairing with RelA/p65) enter the nucleus and bind to target gene κB sites. The non-canonical NF-κB pathway, (right); is activated by members of the TNF-R family (e.g., LTβR, BAFFR, CD40, RANK) by NIK mediated activation of an IKK kinase complex which in turn phosphorylates p100-NF-κB2 leading to its limited proteolysis to produce the p52 form of NF-κB2. Entry of p52-NF-κB2/RELB heterodimers into the nucleus follows, regulating target gene expression. AgR (antigen receptor), LTβR (lymphotoxin β receptor), BAFF/R (B cell activating factor), RANK (receptor activator for NF-κB).

Two disparate but interactive branches of the NF-κB signaling pathways are activated by different initiation signals and control NF-κB activity (Figure 3) [47,48,49]. The IKKβ-dependent canonical (classical) pathway regulates c-Rel, RelA and NF-κB1 (p105/p50) homodimers and heterodimers by phosphorylating members of the IκBα and promoting their degradation [73]. The non-canonical (alternative) pathway, on the other hand is activated by NF-κB Inducing Kinase (NIK) and IKKα phosphorylating NF-κB2 leading to its partial proteasomal degradation which halts at the GRR motif [74] (Figure 1) and leads to nuclear translocation of the NF-κB2(p52)/RelB dimer. Mouse models in which the core components of the NF-κB pathway have been genetically manipulated/deleted have revealed some of the complexities of NF-κB signaling [75]. Stimulation with various agents (Figure 3), such as bacterial endotoxins or the pro-inflammatory cytokine TNF connect through their respective Toll-like receptors (TLRs) or Tumor necrosis factor receptor (TNFR) receptors on cells, including within the PDAC microenvironment with different receptor-proximal signaling cascades and to the IKK complex. The role of TLRs in PDAC has recently been reviewed [76], therefore we will use TNF signaling as an example for the canonical NF-κB pathway, since TNF is also a key inflammation regulator for PDAC (reviewed [77]).

TNF binding promotes recruitment of TRADD and RIPK1 to the receptor and these can in turn recruit TNF receptor-associated factor (TRAF2) [78]. TRAF2 is likely constitutively bound to a pool of the E3 ubiquitin ligases/inhibitor of apoptosis proteins 1 and 2 (cIAP1/cIAP2). TRAF2 binding to the receptor activates them and they ubiquitylate components within TNFR1, most notably RIPK1. The ubiquitin chains are recognized by a TAB/TAK1 complex, LUBAC and to a lesser extent the IKKα/β/NEMO complex. However, M1 linked ubiquitin chains generated by LUBAC are a much more potent recruitment signal for the IKK complex [79,80,81]. IKK2 is most likely phosphorylated and activated by TAK1 [82] whereupon it phosphorylates IκBα. This phosphorylation is recognized by the SCF^βTrCP^ E3 ligase which generates K48 linked ubiquitin chains that are potent signals for recruitment to and degradation by the proteasome (Figure 3).

TLRs, IL-1Rs, RIGI, NOD, BCRs and TCRs all use broadly similar signaling cascades [57,83,84]. Naturally the components are not necessarily the same, but they perform similar functions. Thus, in IL-1R and TLR signaling, TRAF6 substitutes for cIAPs, Interleukin-1 Receptor Associated Kinases (IRAK1-4) substitutes for RIPK1 and other kinases, such as TBK1 are involved in activating IKK2.

Although it is convenient to think of two separate NF-κB pathways in reality they are significantly interwoven (Figure 3). Intriguingly, cIAPs are required for canonical signalling from receptors such as TNFR1 and TCR and are also required within a TRAF2/TRAF3 complex to inhibit non-canonical NF-κB by continuously promoting, NIK ubiquitylation and proteasomal degradation, such that NIK is usually undetectable. Thus, non-canonical NF-κB activating signals work by promoting degradation of cIAPs or TRAFs which allows NIK levels to rise, leading to its auto-activation and phosphorylation of IKKα, which in turn phosphorylates NF-κB2/p100. This is one reason that the non-canonical NF-κB pathway takes longer to activate transcription because it requires production and accumulation of the NF-κB Inducing Kinase (NIK). Furthermore, stimuli that promote non-canonical NF-κB activation by depleting cIAPs or TRAF2 simultaneously reduce the capacity of a cell to respond via the canonical pathway. Again, underlining the links between canonical and non-canonical signaling, NF-κB2/p100 may also bind to p65/p50 and therefore degradation of the p100 ankyrin repeats that occurs in a NIK dependent/non-canonical fashion may result in translocation of the classic, canonically activated p65/p50 dimer [85].

Since NF-κB has the ability to influence the expression of multiple genes, it is not surprising that dysregulation of NF-κB transcription has been linked to the pathogenesis of inflammation, autoimmune diseases and cancers as we [86,87,88] and others have shown [40,41,61,89,90,91]. During inflammation, pro-inflammatory cytokines drive the activation of NF-κB, which perpetuates the inflammatory response [89,92]. Pertinently, PDAC has also been associated with dysregulation of the canonical/non-canonical NF-κB pathways [12,43,51,52,53,93,94,95].

## 3. NF-κB and Pancreatic Inflammation

Increased susceptibility to diseases, including cancer are associated with mutations in the NF-κB signaling pathway [42,49,96,97], while spontaneous mutations in the upstream activators and individual subunits of NF-κB are rare [96], they are more common in diseases of immunodeficiency [98,99]. Our own studies [88,100] and those of others further associate certain functional polymorphisms particularly in NFKB1 to increased susceptibility for various cancers [101]. However, it is more likely that persistent NF-κB activation promoted by pro-inflammatory stimuli underlies disease causing inflammation. Since as discussed, NF-κB can be activated by so many different signals, the inflammatory source may be capricious (viral, bacterial, alcohol, obesity, smoking, cellular senescence, immune activation by malignant cells, or DAMPs (Damage Associated Molecular Patterns released from dying cells) but all are associated with the early events in PDAC development [7,12,14,102].

### 3.1. Pancreatitis Elevates the Risk for PDAC Development

Inflammation is a key feature of exocrine pancreatitis (acute or chronic) [102,103] due to sterile damage; low pH, premature activation of digestive precursor enzymes, such as cathepsin-B, which converts inactive trypsinogen to trypsin activating NF-κB [102,104] and acinar cell death. Repeated episodes of acute pancreatitis can induce the chronic disease; fibrotic tissue and activation of myofibroblast-like cells pancreatic stellate cells (PSCs), with loss of pancreatic function [102,105]. Chronic pancreatitis is a PDAC precursor lesion [26], associated with excess alcohol consumption [21,102,106] and with a 13.3-fold increased risk for PDAC [103,107]. Persistent NF-κB activation is associated with chronic pancreatitis in patients, by promoting low-grade inflammation and creating an opportune environment for tumorigenesis [77,108].

### 3.2. NF-κB a Key Initiator of Inflammatory Mediators in the Pancreas

**Figure 4:**
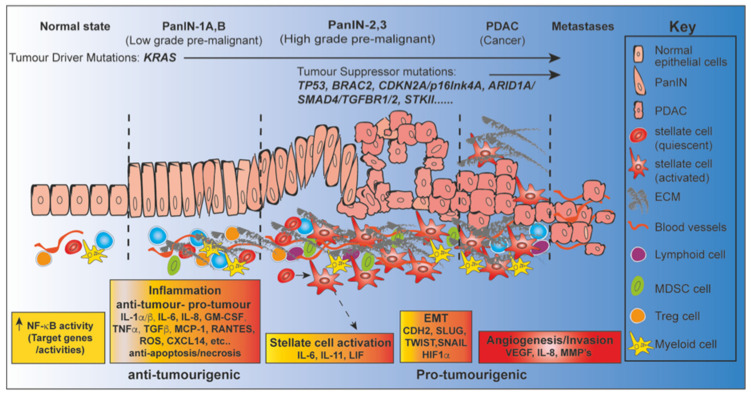
The role of NF-κB from Pancreatic Ductal Adenocarcinoma (PanIN) to Pancreatic Ductal Adenocarcinoma (PDAC) in a step-wise model.

Evidence implicating NF-κB activation in human pancreatitis is supported by animal models [109] and extensively studied [110,111], which we will discuss later in this review. Inflammatory mediators released by dying acinar cells [105] can induce NF-κB activation [112]. Sustained NF-κB induction plays a key role in tissue repair, eliminating injured acinar cells and enhancing stroma formation. Activation of NF-κB, particularly canonical signaling [42] also leads to an influx of inflammatory cells and macrophages, controlled via mediators such as cytokines and chemokines, including TNF [12,42,43,51,52,94]. It is not clear how inflammation promotes PDAC initiation and progression but persistent unresolved acinar cell injury and excessive production of proinflammatory mediators seem to be key [105] (Figure 4). To date, no clinical therapies exist which can reverse the inflammatory damage associated with chronic pancreatitis.

The normal ductal epithelium (left) progresses to PDAC (right), through a series of histopathological precursors (PanINs). During early disease stage the immune system is activated by inflammatory stimuli, resulting in both pro-tumor and anti-tumor effects mediated by inflammatory cells. The NF-κB pathway is dysregulated within the cells of the inflammatory microenvironment and NF-κB secreted target genes serve as fuel (boxes: graded yellow to red) to control both apoptosis and inflammation. Pro-Inflammatory NF-κB target gene mediators can also trigger activation of pancreatic stellate cells (PSCs), promoting neoplastic proliferation, tissue remodeling, such as epithelia-to–mesenchymal transition (EMT), angiogenesis and metastases. Low-columnar epithelium transitions to tall columnar (PanIN-1A, basally located nuclei) to pseudostratified (PanIN-1B), to full thickness (PanIN-2A, nuclear pseudo-stratification) and finally budding into the ductal lumen (PanIn-3, loss of epithelial polarity). During this progression, several alterations in key genes also accumulate: point mutations in *KRAS* occurs during early PanINs, inactivation of *P16* at the intermediate stage and inactivation/mutation of other tumor suppressor genes (*TP53*, *BRCA2*) occurs in later PanINs. Abbreviations, ECM; extracellular matrix, MDSC; myeloid-derived suppressor cells.

### 3.3. NF-κB Promotor or Inhibitor of Pancreatitis?

Agents known to cause human pancreatic inflammation such as alcohol can sensitize pancreatic acinar cells to inflammatory responses. These enhance the effects of cholecystokinin (CCK) on NF-κB activation [113] and in vivo, in combination with bacterial LPS, can result in acute pancreatitis, atrophy and activation of PSCs [114]. Cerulein, is a small, 10 amino acid peptide that was first isolated from the skin of the Australian green tree frog (*Litoria caerulea*) [115]. Like CCK, this CCK analogue stimulates, among other things, secretion of digestive enzymes by the pancreas and is therefore widely used to cause and model acute pancreatitis in mice.

While it was first thought that cerulein induced intra-acinar activation of trypsinogen directly causing pancreatitis, more recent studies suggest the pathogenesis of the disease is not so simple [116]. While trypsinogen activation is a necessary first step, the sustained immuno-pathogenesis of pancreatitis appears to be dependent on the innate immune system. Following acinar cell injury and damage induced release of DAMPs, DAMP-mediated cytokine production permits the movement of gut commensals into the circulation, resulting in innate acinar immune responses and to further NF-κB activation. The resulting increase in the production of inflammatory cytokines (IL-6, IL-1β and TNF) and eventually also IL-13 and IL-33 culminate in the characteristic fibrosis of chronic pancreatitis [111]. This theory was confirmed within the limitations of an acute necrotizing pancreatitis model, in which the hallmark of acute pancreatitis (protease activation) was not restricted to acinar cells but included phagocytic macrophages in both mice and humans. The active trypsin within phagocytosing macrophages acted as the DAMP to fuel systemic inflammation by direct NF-κB (p65/RelA) activation through NLRP3 inflammasome activation [104]. Pancreatitis is therefore a distinctive form of inflammation, in which NF-κB plays a central role and may therefore be amenable to biologic therapies, such as inhibitors of cytokines or their receptors (e.g., anti-IL-6, IL-1Rα, anti-TNF) [117].

Disregarding the exact nature of the stimulus, cerulein undoubtedly induces NF-κB activation, with nuclear translocation of p65/p50, p50/p50 and p52 in pancreatic acinar cells [110,118,119,120]. This activation is inflammatory and accompanied by the transcriptional release of multiple NF-κB target genes, including TNF and MCP-1 [121,122]. A study investigating the role of individual NF-κB components in pancreatitis, established that mice lacking p105/NF-κB1, the precursor of p50, were refractory to this mode of pancreatitis induction [123]. However, this could be due to its role in stabilizing Tpl2 [124], a Map3 kinase activator of Erk signalling in cells [125] and/or the dual role of p50 as a transcriptional activator or repressor [101].

A number of murine pancreatitis models, centered around the use of cerulein, suggest that NF-κB activation in the pancreas is damaging [102,111,126], due to constitutive acinar cell canonical NF-κB activation targeting IKK2 (IKKβ) [127] or nuclear translocation of p65/RelA [43,128]. Similarly, a p65/RelA transgene driven by the acinar-cell–specific promoter (LSL-p65/RelA), increased NF-κB pathway activation in response to cerulein and aggravated cerulein induced pancreatitis [112]. Additionally, in line with the idea that NF-κB can promote pancreatitis, acinar-specific expression of IKK-CA (LSL-IKK2/Cre) resulted in an aggressive spontaneous pancreatitis [112,127]. Furthermore, acinar cell specific ablation of p65/RelA (*rela^Δpanc^*) was found to be protective [126,129].

However, and in contrast, transgenic mice with a deletion of Iκ Bα which also leads to constitutive p65 mediated NF-κB activation, attenuated cerulein pancreatitis. With additional deletion of p65 this safeguard was lost, suggesting NF-κB activation within the pancreas was protective [130]. The apparent inconsistencies in these studies could reflect the differing pathways through which NF-κB activation regulates inflammatory pancreatitis, through induction of inflammatory cytokine mediators or limiting pancreatic inflammation by preventing apoptosis/necrosis (reviewed [126]). Since the IKKs have targets in addition to the NF-κB pathway, their influence may be independent of NF-κB. The take home conclusion is that acinar cell NF-κB activation can trigger both pro- and anti-inflammatory pathways, in a tissue and disease stage specific manner [126].

While it is important to understand the roles of specific NF-κB genes in particular cell types such as acinar cells in pancreatitis the inferences that can be drawn as to the role of these factors in disease progression and the clinical application of this knowledge is complicated by the fact that NF-κB activity is so ubiquitous. Thus, studies addressing the role of NF-κB only in acinar cells [112,130], do not take into account the contribution of NF-κB in immune cells, including myeloid and PSCs in pancreatitis and these may well be opposite roles to those described for acinar cells [104,131]. For example, a mutant IκBα capable of blocking NF-κB activation in mesenchymal stromal cells in a chronic pancreatitis model showed that proliferation and apoptosis of PSCs was regulated by multiple signal transduction pathways (PPAR, MAPK, mTOR, TGF-β, NOD-like receptor, Notch, WNT, TGF-β1-SMAD-2/3, and P53) [132]. Pancreatitis is therefore likely mediated by the crosstalk of intrapancreatic protease activation and inflammation mediated by NF-κB activation within macrophages through pro-inflammatory cytokines, such as TNF (reviewed [76]). In conclusion, pancreatitis initiation, the chronic form and even PDAC development occur through a series of stages [102] and the role of NF-κB at each stage and indeed each cell type (not recapitulated by all pancreatitis models) might be disparate, a point we return to later in this review.

### 3.4. Beyond Inflammation; Multiple Roles for NF-κB in Pancreatic Tumorigenesis

While the role of NF-κB in pancreatitis has been studied, it remains unclear how inflammation promotes PDAC initiation and progression [105]. Indeed, given its wide-ranging roles in proliferation, inhibiting apoptosis and angiogenesis, it may promote PDAC initiation and progression in multiple ways. In addition to these well-established activities, chronic inflammation and NF-κB may also promote chromosomal instability and aneuploidy [42]. Inflammatory cytokines also produce reactive oxygen species, which randomly oxidize DNA to cause genetic mutation. NO induced by inflammation also inhibits DNA repair enzymes to promote mutations. Indeed, the duration of chronic pancreatitis correlates positively with the incidence of KRAS mutations, suggesting that DNA damage accumulates due to the persistence of inflammation, promoting further carcinogenesis [26]. In addition, NF-κB function can be context-specific within the tumor microenvironment, showing variation not only when dysregulated in different cell types but also depending on the stage of malignant transformation (Figure 4). The detailed role of the individual NF-κB transcription factors RelA/p65, RelB, and c-Rel in PDAC development and maintenance has recently been extensively reported [53]. The role of the innate immune NF-κB pathway in relation to PDAC has also been recently extensively reviewed [76]. Therefore, we will briefly review the contribution of these NF-κB elements but focus on other developments in field relating to the transition from inflammation to PDAC.

*KRAS* and *TP53* mutations are found in 20–25% to ~50% of all cancers, respectively [133] and for PDAC in ~90% of patient tumors [13,22,134]. Notably, oncogenic KRAS signaling is an important driver of NF-κB activation in PDAC [50]. An intricate crosstalk between these two pathways results in the constitutive activation of canonical NF-κB through PI3K-AKT-mTOR mediated effector phosphorylation of IKK and activation of p65/RelA (reviewed [76]). More recently, additional signaling pathways resulting in NF-κB activation have been linked to KRAS driven PDAC. For example, IRAK4, which is usually activated by TNF, IL-1R and TLR signalling, has been implicated in PDAC [135] and p-IRAK correlates with poorer patient outcomes. Furthermore, suppression of IRAK4 activity either by genetic or pharmacological methods, reduces NF-κB activity and suppress PDAC growth in vivo [76,136]. TPL2, which is activated by IKK2/IRAK4 signalling, was also identified as driver of an inflammatory autocrine Kras-driven IL-1β signaling loop [137] and activating IRAK4 [135,136]. Thus, TPL2 or IRAK4 inhibition, most likely in conjunction with chemotherapy could be considered as additional therapeutic targets in Kras mutation driven PDAC [135,136]. It is worth noting that TPL2 inhibition also suppresses p105/p50 NF-κB activation [75], providing greater specificity to inhibitors, such as the TPL2 inhibitor GS-4875, currently being trialled for ulcerative colitis (ClinicalTrials.gov Identifier: NCT04130919).

Two kinases: IKKε and TANK-binding kinase 1 (TBK1), while not part of the classical IKK signaling complex, can nevertheless activate NF-κB and upregulate type I interferon genes [138,139]. Elevated TBK1 activity has implicated in the development of several cancers [140,141,142] and elevated expression is associated with *KRAS* mutations and poor prognosis in PDAC [140]. Genetically engineered mouse models (GEMMs) have been developed to mimic the traditional step-wise accumulation of mutations in genes such as *KRAS* in the human pancreas [134] (Figure 4). Typically, these models have pancreas-specific expression of a mutated KRas protein, such as KRas^G12D^ [143,144]. When *Trp53* or *Cdkn2a* defects or administration of cerulein are also introduced, the disease is accelerated [145], suggesting that additional abnormalities, such as inflammation are required for disease progression, as for the human disease [146,147]. Just such a GEMM was used to explore the involvement of TBK1, where it was shown that mice with the *Tbk1^∆/∆^*; *Kras^LSL-G12D^*; Cdkn2a^lox^*^/lox^*; Ptf1a^Cre^*^/+^*, genetic background had elevated autophagic markers in PDAC tissues [140]. Similarly, a link between autophagy inhibition and TBK1 signaling in the cerulein-induced pancreatitis model has been described [148]. Together, these studies indicate that TBK1 may contribute to the survival of PDAC by regulating autophagy. This link also includes RalB a monomeric RalGTPase, which recruits and activates TBK1 and its effector protein sec5 in a RalB effector complex, reviewed [139]. In tumor cells the RalB-Sec5-TBK1 pathway inhibits apoptosis and perpetuates cancer cell survival [149]. We therefore discuss the use of TBK1 inhibitors [142] in the context of PDAC later in this review (Section 6.4.3)

Constitutive activation of the alternative NF-κB pathway by NIK is involved in the proliferation of pancreatic cancer cells [150]. As discussed earlier, NIK expression can result from loss of TRAF2, and its activity in most PDAC cell lines is maintained by proteasomal downregulation of TRAF2, and reduction in TRAF2 levels correlates with increased aggressiveness of human tumors [150,151,152,153]. PDAC cell lines with elevated NIK expression have elevated levels of NF-κB2 target genes; CCL19, CCL21, CXCL12, CXCL13 and BAFF, implying that activation of the NIK/NF-κB2 pathway could drive rapid and aggressive PDAC growth [152,154]. Oncogenic *KRAS* also promotes constitutive NF-κB activation by inducing glycogen synthase kinase 3α (GSK-3α). GSK-3α binds to the IKK-activating protein complex, consisting of transforming growth factor-beta activated kinase 1 (TAK1) and TAK1-binding protein (TAB), and stabilizes it, thus enhancing canonical NF-κB activation. GSK-3α also appears to play a role in activating the non-canonical NF-κB pathway because p100 processing was reduced by GSK-3α knock-down although the mechanism for this was not explored [155].

Just as with pancreatitis, GEMMs have been used to determine the function of IKK complexes (IKKα or IKKβ) during tumorigenesis and have revealed a high complexity in NF-κB signaling. Similar to other tumors, the constitutive activation of NF-κB in PDAC is primarily determined by pro-inflammatory cytokines, such as TNF and IL-1α released by tumor-infiltrating immune cells. For example, pancreas specific deletion of *Ikbkb* (IKKβ) in the *Kras^G12D^* PDAC model showed a delayed phenotype with lower grade lesions, partially dependent on IL-1α and NF-κB activation [156,157]. Studies also demonstrated that IL-1α drives the activation of NF-κB in PDAC cell lines, establishing a positive feedback loop for constitutive NF-κB activation [156,158]. Increased expression of protein kinase D1 (PRKD1) is also linked to oncogenic signaling in PDAC. The PRKD1 gene promoter is a target for oncogenic KRas signaling and activation of the canonical NF-κB pathway. Binding sites for NF-κB also within the PRKD1 promoter, provides a further functional link between oncogenic KRas, NF-κB and expression of PRKD1 [159].

## 4. The Role of NF-κB in Various Facets of PDAC Biology

One of the main therapeutic barriers in PDAC is the unique desmoplastic stroma consisting of a dense extracellular matrix (ECM) infiltrated with a mix of cancer-associated-fibroblasts (CAFs), immune and endothelial cells [160] (Figure 4). Interlocking pathways in the stroma promote invasive behaviour such as epithelial-mesenchymal transition (EMT), which is promoted by the stroma, excellently reviewed [160]. Epithelia-to–mesenchymal transition (EMT) occurs at very early stage in PDAC progression, before emergence of frank tumorigenesis, precipitated predominantly by inflammation and locally elevated cytokine production [161]. NF-κB signaling makes a major contribution to the desmoplastic stroma [160]. For example, CAFs secrete chemokines and cytokines that enhance tumor progression and therapeutic resistance and their transition between states is modulated by NF-κB. The contribution of CAFs and other immune cells and their modulation by NF-κB in the context of PDAC is reviewed later in this section.

### 4.1. Cell Death; Apoptosis and Autophagy

Apoptosis plays critical roles in normal development, tissue homeostasis and immunity, and its dysregulation contributes to many pathologies, including cancer [162]. Apoptosis is triggered either by engagement of death receptors of the TNF receptor family on the cell surface or by diverse intracellular signals that act upon the Bcl-2 protein family, which controls the integrity of the mitochondrial outer membrane. Activation of either the intrinsic or extrinsic apoptosis pathway leads to cellular demolition by caspase proteases [162]. NF-κB regulates many pro-and anti-apoptotic genes in both the extrinsic: *cIAPs*, *Trail*, *caspase-8*, *c-Flip*, and intrinsic: *Bcl-2* and *Bcl-X_L_* [133,163] pathways, with NF-κ activation often tipping the balance in favour of anti-apoptotic genes such as the caspase-8 inhibitor Flip or the IAP proteins (c-IAP1/2 and XIAP). NF-κB and p53 antagonistically regulate each other’s activity, with IKKβ directly phosphorylating p53 to promote its ubiquitylation and degradation [42]. p53 can also activate transcription of the pro-apoptotic proteins Puma and NOXA [164]. In contrast to other cancers, there have been reports that the anti-apoptotic protein Bcl-2 is not generally overexpressed [165] and, surprisingly, low expression may be correlated with poor prognosis [166]. IAPs, such as XIAP and cIAP are also overexpressed in pre-cancerous and invasive PDAC [167] and this NF-κB mediated mechanism contributes to PDAC’s chemo and radio-resistant resistance to apoptosis [167]. Modes of combating such resistance through IAPs are discussed later [168].

Deregulation of the NF-κB pathway and macroautophagy (autophagy) is frequently observed in cancer cells, serving both pro-tumorigenic and suppressive roles and resistance to cancer therapy [169]. Autophagy occurs when autophagosomes deliver cytoplasmic constituents to lysosomes for degradation [170,171] and is involved in several cellular functions regulated by NF-κB, including cell survival, differentiation, senescence, inflammation, and immunity (reviewed [172,173,174]). PDAC has a distinct dependence on autophagy (recently reviewed [169]) and genetic or pharmacologic inhibition of autophagy results in significant growth suppression of PDAC cells in vitro and in vivo [175,176]. As discussed, a major route in the development of PDAC is via acinar cell damage and dysfunction and one reason that autophagy might contribute is that it is required for the maintenance of acinar cells [169] and at early disease stage (during the development of inflammation/pancreatitis [105,177]). Autophagy and NF-κB share common upstream signals and regulators and can control each other through positive or negative feedback loops to regulate each other’s activity [163,172,178,179]. For example, activation of p65/RelA can upregulate expression of autophagy proteins, such as beclin 1 and SQSTM1 [50] (recently reviewed [174]). Additional studies have shown that induction of autophagy mediated by OPN (oestopontin)/NF-κB signaling is required for maintenance of PDAC stem cell activity and combination of gemcitabine with pharmacological autophagy inhibitors (NF-κB inhibitors) could be a promising therapeutic strategy [176,180].

### 4.2. NF-κB and Angiogenesis

Inhibition of angiogenesis is a promising therapeutic approach for cancer. However, several recent reports suggest that activation of NF-κB is a common mechanism of both angiogenesis and angiostatic agents, resulting in both stimulation and inhibition of angiogenesis [181]. One of the earliest events in angiogenesis is the degradation of the vascular basement membrane and the remodeling of the extracellular matrix [181], which is fueled by angiogenic factors; vascular endothelial growth factor (VEGF), fibroblast growth factor (FGF) and platelet-derived growth factor (PDGF) and IL-8, all of which are NF-κB target genes. NF-κB signaling blockade can inhibit in vitro and in vivo expression of VEGF, IL-8, and MMP-9, resulting in decreased neoplastic angiogenesis [182] and metastasis of pancreatic cancer (Figure 4) [183]. Suppression of NF-κB activity has also been attributed in part to reduce PDAC growth through angiogenic inhibition [184].

### 4.3. Metastasis and NF-κB

As discussed PDAC often presents late, when the tumor has already metastasized [6]. Metastasis requires an Epithelial–Mesenchymal Transition (EMT) [185], a process coordinated by a series of transcription factors (EMT-TFs), notably *CDH2*, *SNAIL*, *SLUG*, *TWIST*, and *ZEB1* [186]. NF-κB promotes EMT migration and invasion of pancreatic carcinoma cells (Figure 4) [187]. Inhibition of NF-κB has been shown to impede the transcription of *CDH2*, *SLUG*, *TWIST1* and *SNAIL* in some cancer cell lines [188,189,190,191] and NF-κB activation by TNF or expression of constitutively active IKK2 can induce an EMT-phenotype [187]. In addition to regulating EMT genes, NF-κB can promote metastasis through HIF1α, by enhancing hypoxic conditions and survival of metastasis-triggering cells [192]. Additional NF-κB target genes, useful at later steps in the invasion-metastasis cascade, such as the matrix metalloproteinases MMP-2, -3, and -9 can also enhance tumor cell intravasation and extravasation [186].

### 4.4. NF-κB and Chemotherapeutic Drug Resistance

Drug resistance is a major obstacle causing treatment failure and resulting in poor survival for PDAC. NF-κB is the main culprit in the acquired resistance against gemcitabine, the standard treatment for locally advanced and metastatic PDAC [193]. Multiple aspects of NF-κB pathway signaling outcomes impact on acquired resistance against gemcitabine, platinum agents and topoisomerase inhibitors, part of the FOLFIRINOX regime [31,194,195]. Inhibition of NF-κB subunits, particularly silencing of RelA/p65 with siRNA has been shown to sensitize gemcitabine-sensitive PDAC cells to gemcitabine, although it had no effect on gemcitabine-resistant cells [196]. In another study, silencing of RelA/p65 synergized with gemcitabine to inhibit the proliferation and induce PDAC cell apoptosis in vitro and in vivo in murine models [197]. Cross talk of NF-κB with other pathways also leads to gemcitabine resistance [193,198,199] and reviewed [50]. Thus, simultaneous targeting of NF-κB signaling pathways along with the chemotherapeutic agents may have potential to improve outcomes [95].

## 5. An Emerging Role for NF-κB in the Tumor Microenvironment

NF-κB is expressed not only in malignant pancreatic cells and normal cells but also a multitude of types constituting the tumor’s milieu. These heterogeneous cell populations and their interactions with tumor stroma contribute to PDAC progression. PDAC itself is poorly vascularized with an extensive stroma that is composed of Cancer Associated Fibroblasts (CAFs), immune cells; T regulatory cells (Tregs), immature monocytes, dendritic cells, mast cells, NK cells, neutrophils, PSCs, tumour-associated macrophages (TAMs)) and endothelial cells [200,201,202] (Figure 4). The microenvironment is also biased towards an immunosuppressive phenotype due to M2 polarized macrophages, Tregs and immature myeloid derived suppressor cells (MDSC) (reviewed [77]). Of the inflammatory cytokines mediating the crosstalk between tumor cells and immune cells, TNF appears to play a pivotal role [77]. Clinical trials for PDAC have focused on the targeting of the tumor cells themselves, rather than microenvironment constituents, such as TNF or the factors controlling its production such as NF-κB. A better understanding of these networks is required to develop more powerful treatment strategies [203]. However, determining the physiological role of individual NF-κB dimers themselves using gene knock-out mice is complicated and the combinatorial diversity of the NF-κB dimers adds to their ability to regulate distinct but overlapping sets of genes by influencing various aspects of κB site selection [41,47,48].

### 5.1. NF-κB and Myeloid Subsets in PDAC

Distinct myeloid subsets have been correlated with tumor promotion and anti-tumor immunity. TAMs in response to microenvironmental signals, can influence tumor progression by releasing growth factors orchestrating enhanced inflammation, inhibition of immune surveillance and angiogenesis [204]. Elevated TAMs are associated with chemoresistance and poor patient outcomes in many solid cancers and this is also the case for PDAC [202,205,206]. TAMs could therefore be considered as a viable therapeutic target. Plasticity is a hallmark of mononuclear phagocytes and macrophages. They exist in various polarized states in response to environmental cues: quiescent macrophages (M0), classically activated (M1) and alternatively activated (M2). TAMs of multiple types of solid tumors have a functional phenotype akin to M2 macrophages [204] and NF-κB plays a role in finely tuning their functions. Accumulating evidence exposes a crucial role of TAMs in PDAC progression. Myeloid cells lacking p50 have a proinflammatory phenotype [207,208] and recent studies have shown that 5FU followed by reconstitution with *Nfkb1*^−/−^ immature myeloid cells slowed the growth of PDAC in the *K*-*Ras^G12D^* PDAC model, in a CD8^+^ T cell dependent manner. This suggests that adoptive therapy of immature myeloid cells may also be possible for PDAC patients [209]. Myeloid cells also appear to support immune evasion in PDAC through EGFR/MAPK-dependent regulation of PD-L1 expression on tumor cells, since depletion of myeloid cells prevented *Kras^G12D^*-driven PDAC initiation and restored CD8^+^ T cell anti-tumor immunity [210]. This finding has implications for the design of immune therapies for pancreatic cancer, centered around myeloid cell control and there are number of ongoing clinical trials for PDAC targeting TAMs (reviewed [202]).

### 5.2. NF-κB and Pancreatic Stellate Cells (PSCs)

Pancreatic Stellate Cells (PSCs) are a pluripotent cell type located between pancreatic lobules and acinar cells [211] that in a healthy pancreas are quiescent. In response to pancreas damage and inflammatory signals, PSCs become activated, proliferate, and can differentiate into myofibroblast like cells that contribute to repair via a fibrotic process involving the production of large amounts of extracellular matrix components. PSCs also play a pivotal role in PDAC progression where, likely stimulated by the inflammatory environment they can generate CAFs. The fibrotic process can contribute to metastasis and serves as a barrier to chemotherapeutic drug delivery [200,211,212].

This simple picture [213] is however complicated by recent studies using co-cultures of murine PSCs and metastatic pancreatic organoids showing that PSCs in cancers are not a single entity but rather four sub-populations, resting PSCs and three mutually exclusive and reversible CAF populations, with distinct spatial and phenotypic roles [214,215]. Myofibroblastic cancer associated fibroblasts (myCAFs) are defined by high αSMA and do not produce inflammatory cytokines. The inflammatory, induced CAFs (iCAFs), on the other hand, are promoted by NF-κB and characterized by reduced αSMA and secretion of cytokines and chemokines such as IL-6, IL-11 and LIF. Yet, a third type of “antigen-presenting CAFs” has been identified that express MHC class II and CD74 without classical costimulatory molecule expression and which may therefore act as a decoy to deactivate CD4^+^ T cells and thus modulate the immune system [216]. The nuclear levels of RelA/p65 are elevated in iCAFs compared to resting CAFs/myCAFs and support the idea that NF-κB signaling is required for the formation of iCAFs, through activation by IL-1α [215]. IL-1α has been suggested as the missing link between *Kras* activation and constitutive NF-κB activation [156]. The detailed mechanisms governing CAF formation, their transition between states and how NF-κB modulation influences this, still requires further investigation as does the view that all cells within the tumor stroma are pro-tumorigenic [217]. There also appears to be support for the view that CAF type can be manipulated. For example, the secretory phenotype of iCAFs suggests they play a role in promoting tumor progression and immune suppression, therefore, depleting them or shifting them to a myCAF phenotype could reduce secretion of tumor promoting cytokines which may restrain tumor suppression. Studies of GEMM mice and cell lines suggest that reduction in p50 NF-κB activity in PSCs may reduce PDAC [218]. In this study, it appears that p50 NF-κB activity in PSCs promoted tumor growth by increasing expression of CXCL12 and preventing cytotoxic CD8^+^ T cells from infiltrating the tumor and killing PDAC cells [218]. These findings underline possible clinical implications of CAFs as biomarkers and as potential targets for prevention and treatment of PDAC [219].

### 5.3. NF-κB and Tregs

In general, Tregs in the tumor microenvironment can inhibit anti-tumor immune responses [220]. Key roles for NF-κB subunits, p65 and c-Rel have been established for CD4^+^ Foxp3^+^ Treg identity and function [60,221,222,223,224]. While an exact role for Tregs in the PDAC microenvironment has not been defined (reviewed [77]), they are abundant in PanIN and pancreatic cancer and may work by suppressing tumor-associated DC immunogenicity [225]. Recently this theory was tested using a modified *Kras*^+/LSL–G12D^ GEMM that specifically expresses the Diphtheria Toxin Receptor (DTR) in Tregs (KC;*Foxp3*^DTR^). Injection of Diphtheria Toxin into mice, which do not otherwise express the DTR, specifically kills Tregs and in this *Kras*^+/LSL-G12D^ PDAC model, completely counter to the immune suppression hypothesis, actually accelerated PDAC progression [226]. Tregs are a major source of TGFβ ligands and, possibly as a consequence of this, their depletion resulted in fibroblast reprograming and conversion from SMA^high^ myCAFs to immunosuppressive TAMs [226] (as described in Section 5.2). This unexpected complex exchange between Tregs and fibroblasts in PDAC, exposes CCR1 blockade as a novel therapeutic approach to prevent immunosuppression in PDAC [226].

Tumor models of other cancers have shown that loss of Treg expression of c-Rel, but not p65 slowed melanoma growth as did chemical inhibition of c-Rel function, with targeting of c-Rel also enhancing checkpoint-targeting immunotherapy for this cancer [227]. A protocol, of immunotherapy (immune checkpoint inhibitors (e.g., anti-PD-1, -PD-L1 or -CTLA4) in combination with NF-κB inhibition or Treg depletion [225] perhaps should be considered to circumvent T cell exhaustion [36,37].

To summarize this section, NF-κB in the PDAC microenvironment is complex but overall seems to promote tumor growth and chemo-resistance and suppress an anti-tumor immune response. Therefore, the micro-environment is potentially a source of targetable NF-κB vulnerabilities, and an immunotherapeutic “Achilles heel” [219,228]. Treatment targets based on modulation of the PDAC microenvironment are now being assessed in combination with standard chemotherapies [50,77,229].

## 6. Inhibiting NF-κB in PDAC; Is Therapy Possible without Unwanted Side-Effects?

PDAC is resistant to all traditional forms of cancer therapies, possibly due to the high abundance of molecular alterations [27,28,30,102,147,230]. Activating KRAS is the most common mutation in PDAC and therefore the most desirable molecular target, however it is currently not druggable. The high prevalence of NF-κB activation in PDAC makes it an attractive therapeutic target and pharmacological targeting of the canonical and non-canonical pathways [33,50,51,52,53], would seem to be a logical alternative approach [231]. This is particularly the case for PDAC, since the most significant issue with treatment of advanced disease is the acquired resistance to gemcitabine, in part mediated by NF-κB [50,193,232]. However, targeting NF-κB is perilous, since this transcription factor is also involved regulating a broad range of normal cellular homeostatic activities [47,56,57,58,59,60] and as it cross-talks with other signaling pathways also deregulated in PDAC (STAT3, Notch and Hedgehog) [157,198,233,234,235,236]. Over 780 inhibitors of the NF-κB pathway have been identified, including compounds [237] and small molecules [238], acting in a broad range or specific manner. However, the vast majority of these compounds are yet to be tested in the context of PDAC [51,231,239] and few are in clinical use [33,50,53,231,239,240]. A summary of the data relating to NF-κB modifying agents, with a particular focus on promising newer candidates, including some of our own SMAC mimetic studies are included in this review.

### 6.1. Generalized Anti-Inflammatories

**Figure 5:**
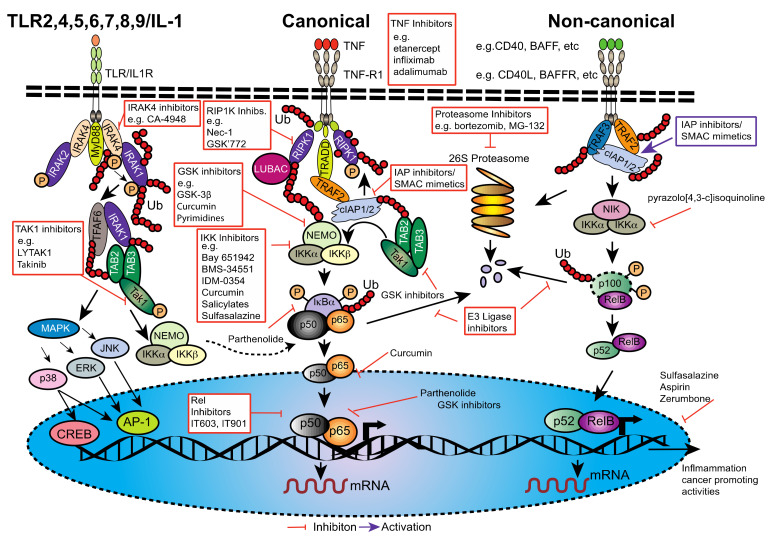
NF-κB; activation and inhibition. Simplified schematic diagram of the canonical and non-canonical NF-κB and TLR/IL-1R pathways. Red arrows indicate intervention points within each pathway for NF-κB and other pathway inhibitors and red boxes list examples of drugs. Many inhibitors are broad range (both pathways), others target a precise stage of NF-κB activation and yet others target multiple stages of NF-κB or TLR/IL-1R signaling.

A variety of mainstream anti-inflammatory drugs agents which also inhibit NF-κB have been tested in PDAC, including glucocorticoids, nonsteroidal anti-inflammatories (NSAIDs) and disease-modifying anti-rheumatic drugs (DMARDs) [51,231]. The classical anti-inflammatory drug aspirin is the most noteworthy (Figure 5) [241]. Although its best understood targets are the COX enzymes, therefore blocking the production of inflammatory prostoglandins and thromboxanes [241], it may also block NF-κB activation by inhibiting IKKβ [242] and cell death by inhibiting caspases [243]. The anti-inflammatory drug sulfasalazine can also inhibit IKK, suppress PDAC growth [231] and sensitizes PDAC cell lines to chemotherapy [244]. Parthenolide, a sesquiterpene lactone, directly binds to and inhibits IκBα and also blocks the binding of NF-κB to DNA [231] (Figure 5). The combination of the COX2 inhibitor sulindac, another NSAID and parthenolide cooperatively mediated growth suppression of pancreatic cancer cells by inhibiting NF-κB [245]. The specific COX2 inhibitor Celecoxib can also reduce activation of NF-κB, when used in combination with gemcitabine, albeit in a pancreatic cell line specific manner [246]. Overall, these studies suggest NF-κB inhibition as a potential target for PDAC prevention and COX2-inhibitors as chemo-preventive agents. However, global blockade of NF-κB activity with anti-inflammatories can result in unwanted side effects including immunosuppression [247].

### 6.2. Curcumin, Flavinoids (Natural Phenolic Substances) and Proteasome Inhibitors

Numerous natural compounds have been documented as NF-κB pathway inhibitors [237] but the majority remain untested for PDAC. One of the few validated compounds is curcumin (diferuloylmethane), the natural pigment found in turmeric [51]. It has been shown to be anti-inflammatory with antioxidant and anti-tumor metastatic activities when used in vitro [188,248]. The proposed mechanism is inhibition of phosphorylation and degradation of IκBα [248,249,250] (Figure 5) or inhibition of the ROS/ERK/NF-κB signaling pathway [188]. It is important to note however that the supra-physiological doses used in these in vitro studies might not be attainable in patients. Nevertheless, a phase 2 clinical trial for PDAC concluded that oral curcumin gave a partial response [251]. Additional trials are reported [252] or underway (ClinicalTrials.gov Identifier: NCT00094445). As described earlier, NF-κB activation requires proteasome activity to degrade the inhibitory IκB molecules (Figure 3). While blocking proteasomal degradation of IκB might have been an impetus for trialing proteasome inhibitors such as bortezomib (Velcade, PS-341) for the treatment of Multiple Myeloma (MM) [93,238,241], the anti-cancer activity of proteasome inhibitors is unlikely to be due only to their ability to block NF-κB activation [253] (Figure 5). Nevertheless, in PDAC cells, the proteasome inhibitor MG-132 has been shown to decrease NF-κB activity and reduce proliferation, invasion and angiogenic potential which correlated with a reduction in VEGF and IL-8 secretion [254,255]. Bortezomib has also been shown to enhance the activity of docetaxel in orthotopic human PDAC xenografts [256]. However, its use in a phase II clinical trial for PDAC, alone or in combination with gemcitabine lacked efficacy and was hampered by toxicity [257].

While Curcumin and bortezomib have some NF-κB inhibitory activity their actions are much more broad, and therefore the remainder of this section will focus on drugs with proven NF-κB specificity.

### 6.3. Direct Inhibition of NF-κB or NF-κB Subunits

Since IκB degradation is induced by IKK phosphorylation (Figure 1 and Figure 3), one strategy to inhibit NF-κB is to block IKK activation or activity. More than 150 agents have been shown to inhibit this step (reviewed [238]), including adenosine triphosphate (ATP) analogs and compounds that interact with a Cys-179 in the activation loop of IKKβ [238]. Proof of principle studies expressing a dominant-negative IKKβ kinase have suggested that IKKβ inhibition has therapeutic potential for inflammatory diseases [258]. Furthermore, a cell-permeable IKKβ peptide has been shown to block the binding of NEMO to IKK [259], implying that it is possible to directly inhibit the pathway with small molecules. However, pharmacological targeting of IKKs comes with the risk of severe side effects arising from global NF-κB inhibition [260,261]. This is mostly likely the reason that IKKα/β inhibitors such as SAR-113945 and MLN-0415 fell short in early clinical trials for osteoarthritis, arthritis and multiple sclerosis (reviewed in [261,262]). In anti-cancer trials IKK inhibitors such as AS-602868, GMX1777 and CHS-828 have not advanced beyond Phase 1/2 [262]. Pertinent to this review, IMD-0354 has been shown to suppress PDAC cell growth in the NOD/SCID/γc^null^ model of orthotopic human PDAC without adverse effects, although doubts have been raised as to whether IMD-0354 is an IKKβ inhibitor [263]. Although, thus far, no IKKα/β or IKKβ inhibitors have been approved for clinical use, their use is still being considered in combination with other therapies to overcome intrinsic or acquired resistance of cancers to chemo and even immunotherapy [261].

Studies in NF-κB knockout mice have shown that this pathway can be selectively targeted and that individual subunits play distinct roles that cannot be fully compensated by other Rel-family members [75]. Targeting the activity of specific NF-κB proteins (or their downstream functions) could therefore be viewed as a superior approach. For instance, elevated RelA/p65 expression is associated with NF-κB activation and its nuclear localization has been reported in PDAC cell lines, patient PDAC samples [156,264,265]. A number of approaches aimed at limiting RelA/p65 activity have recently been reviewed [53] and include the use of p65/RelA siRNA to overcome chemoresistance to gemcitabine or by negative regulation through interfering with post-translational modification mechanisms [238,266].

In contrast to other NF-κB proteins, which are ubiquitously expressed, c-REL is mainly located in mature haematopoietic cells [267] and associated with hematological malignancies. Reports for a role for c-REL in solid cancers are limited [268,269] and few that speak to its role in PDAC [53]. Some progress has been made in developing small molecule inhibitors of c-REL (IT603 and IT901) [270,271,272]. IT603 has been shown to enhance tumor inhibition by anti-PD-1 immunotherapy in a melanoma model [227]. In terms of PDAC, c-Rel has been shown to mediate TRAIL-induced apoptosis by controlling tumor-promoting genes, such as NFATc2 [269,273]. Targeting c-Rel might therefore be worthwhile exploring for PDAC, particularly if next generation inhibitor compounds become available.

While much of the NF-κB-related research focuses on the canonical pathway, the non-canonical NF-κB pathway is also generally dysregulated in many cancers [90] and in PDAC [151]. Targeting elements of this pathway such NIK, which maintains a high basal activity in PDAC [151] or IKKα could be fruitful [93]. However, few NIK inhibitors have been described and those that have, such as the pyrazoloisoquinoline derivative pyrazolo[4,3-c]isoquinoline (Figure 5), have µM IC50s and also inhibit other kinases [93].

### 6.4. Inhibition of NF-κB Signaling Components

#### 6.4.1. IRAK4 Inhibitors

The innate immune signaling pathway plays a pivotal role in inflammation. Activation of TLR/IL1R results in the formation of the Myddosome, a complex consisting of MyD88, IRAK4 and IRAK2 (Figure 5), ultimately resulting in the activation of the IKK complex and NF-κB, particularly in myeloid and lymphoid cells [274]. This pathway is often dysregulated in leukemias and lymphomas resulting in elevated NF-κB activity [274,275]. Several IRAK4 inhibitors have been developed (Figure 5) and are currently being clinically evaluated for inflammatory diseases including PF-06650833, BAY-1830839 and BAY1830839, (reviewed [76,262,276]). In particular, CA-4948 a small molecule inhibitor of IRAK4 is being pursued with an ongoing phase 1/2 study for relapsed/refractory hematologic malignancies (ClinicalTrials.gov Identifier: NCT04278768) [277], although it also inhibits FLT3 [262].

#### 6.4.2. Transforming Growth Factor-β (TGF-β)-Activated Kinase 1 (TAK1) Inhibitors

Diverse ligands including TLRs, IL-1 and TNF activate TAK1 through assembly with the TAK-1 binding proteins (TAB1-3), initiating cellular signaling and activation of NF-*κ*B and MAPKs [278] (Figure 5). TAK1 itself plays a role in the cellular responses to environmental stress during inflammation and is a key mediator in the regulation of cell survival and death in cancers and inflammatory diseases [279]. Targeting TAK1 is therefore of particular therapeutic interest and several novel TAK1 inhibitors have been developed (Figure 5). Takinib is an ~10 nM inhibitor of TAK1 that targets both non and autophosphorylated forms that was well tolerated in vivo over several weeks and which reduced the inflammatory response of mice to LPS injection [280,281]. Another TAK1 inhibitor, LYTAK1 has been shown to inhibit *KRAS* mutant colorectal cancer cell growth both in vitro and in vivo, however in vitro, doses of 50 µM were required [282].

#### 6.4.3. TANK-Binding Kinase 1 (TBK1) Inhibitors

TBK1 is a non-canonical IKK serine/threonine kinase that can phosphorylate and activate p100/NF-κB2. It is involved in multiple signaling pathways including inflammation, autophagy and Ras mediated oncogenesis [140], but is best known as a key player in the induction of type I interferon responses [283]. TBK1 activity is tightly controlled, but when dysregulated it is associated with inflammatory and autoimmune diseases and cancer [284]. This includes PDAC, where it is highly expressed which is in turn correlated with poorer overall survival [140]. This has led to the pharmacological development of many TBK1 inhibitors: MRT67307, AZ13102909, SR8185-related compounds, and several others [285]. As a class these compounds are generally quite specific (although all have activity against the very closely related IKKε), have low nM IC50s and seem to be well tolerated in vivo, although testing has so far been mostly limited to preclinical animal models [140,276,286]. Momelotinib (CYT387) was originally developed as a JAK kinase inhibitor however was subsequently shown to also be a low nM TBK1/IKKε inhibitor [287]. Exploring new avenues for TBK1 targeted therapy in PDAC have shown that Compound II can reduce viability in human PDAC cell lines and reduced tumor burden in a pre-clinical model of KRAS driven PDAC (*p48-Cre*; *LSL-Kras^G12D^*; *Cdkn2a^lox^*^/*lox*^, KIC) [288]. The functional complexity of TBK1, however increases the obstacles pursuing it as a clinical drug target, since prolonged inhibition may expose to increased risk to viral infection [286]. Pertinently to the topic of this review, clinical trials for *KRAS* mutant PDAC with momelotinib, were unsuccessful [140], despite being effective in reducing tumor burden in a *KrasLSL*^G12D/WT^–induced lung cancer model [287].

#### 6.4.4. TNFR Pathway Inhibitors

Dysregulated NF-κB activates cytokines and chemokines, including the master pro-inflammatory cytokine TNF [285]. Anti-TNF biologics, are established therapies for autoimmune inflammatory diseases [117] with five such drugs in clinical use; etanercept, infliximab, adalimumab, golimumab, and certolizumab pegol (Figure 5) [289]. As discussed in this review, TNF is also important at many stages in PDAC development; early inflammation, invasion [290], chemoresistance [291] and endothelial-mesenchymal transition [285]. Early studies with infliximab or etanercept in PDAC were encouraging, reducing tumor growth and metastasis in a murine model of orthotopic human PDAC [290] and with etanercept impairing EMT-associated properties in premalignant human pancreatic epithelial cells in vitro [292]. However, in clinical trials anti-TNFs have been disappointing. The addition of infliximab to gemcitabine to treat cachexia in advanced PDAC was not more efficacious than placebo [293] and combining etanercept with gemcitabine in a phase I/II clinical trial did not enhance the outcome for advanced PDAC over gemcitabine alone [294]. Currently no clinical trials for anti-TNFs in PDAC are underway [295,296].

RIPK1 mediates the responses downstream of TNFR1 signaling and is an important mediator of inflammation and cell death through activation of NF-κB signaling [276,297]. Very specific, low nM inhibitors of RIPK1 have been developed which block TNF induced cell death [298], including Necrostatin-1 (Nec-1), GSK′772, GSK′095, DNL747 and GNE684 (Figure 5), [276,297,299,300]. Since RIP1K inhibitors block the TNF cell death rather than transcriptional response it is hoped they will have advantages over blanket TNFs inhibition [299]. These inhibitors have progressed from pre-clinical models where they have been very successful to human clinical phase I/IIa trials, where they have not performed so well even though apparently well tolerated [299,300,301]. GSK′095 (GSK3145095) was trailed for PDAC and other solid tumors alone and in combination with other anticancer agents [299], following influential publications suggesting that RIPK1 played a role in pancreatic oncogenesis by suppressing an immune response. Unfortunately, the results from these papers have not been reproduced by other authors [298] and the phase Ib/IIa trial (ClinicalTrials.gov Identifier: NCT03681951) was prematurely terminated in 2020 due an internal GSK review.

### 6.5. Other Modes of NF-κB Inhibition and Combination Therapy

Inhibiting NF-κB may, in addition to reducing tumor growth, also sensitize tumor cells to chemotherapy. For example, pomalidomide (Figure 5), a third-generation immunomodulating drug derived from thalidomide, has been shown to promote the chemo-sensitization of PDAC by inhibiting chemotherapeutic agent-induced NF-κB activation [302]. Two downstream effectors of KRAS have been identified as targets for KRAS inhibition, which coordinately regulate NF-κB signaling pathways and may also offer an alternative route to inhibit NF-κB. These are TAK1 and GSK-3α [155] (Figure 5). Pharmacologic inhibition of GSK-3α with AR-A014418 suppressed growth of human PDAC explants and reduced c-Myc and cIAP2 expression. Inhibition of TAK1 expression by modulating GSK3 activity could also represent a valid approach to revert in vivo the intrinsic chemoresistance of PDAC [50,303]. In addition, destabilization of TAK1-TAB by inhibiting GSK-3α could also offer therapeutic opportunities for PDAC [304]. Another small molecule PBS-1086 which has been reported to inhibit both canonical and non-canonical NF-κB showed some efficacy in multiple myeloma xenografts models when combined with bortezomib [305]. While combinatorial targeting of both the canonical and non-canonical NF-κB pathways may act synergistically, it runs the strong risk of adverse events, therefore this approach may be impractical.

### 6.6. Smac Mimetics; The Future for NF-κB Inhibition and PDAC Therapy?

Another potential therapy and associated molecular NF-κB target for PDAC is the inhibition of the mammalian ubiquitin ligases, the Inhibitory Apoptosis Proteins (IAPs; XIAP, cIAP1, and cIAP2) [168]. As discussed earlier, cIAP1/2 participate in positive (canonical) and negative regulation (non-canonical) of NF-κB (Figure 5). This interplay is well illustrated by the action of IAP antagonist drugs (Smac-mimetics) that promote the proteasomal degradation of the cIAPs and thereby allow NIK to accumulate and activate the non-canonical NF-κB pathway. This results within a subset of cells in the production of TNF. Since cIAPs are required for TNF induced activation of the pro-survival canonical NF-κB pathway and to prevent TNF induced death, Smac-mimetics can cause TNF induced apoptosis of a subset of cancers by simultaneously activating non-canonical NF-κB and inhibiting canonical NF-κB [306,307,308].

As discussed, resistance of PDAC to current treatments including radiotherapy remains a major challenge. Increased expression of IAPs is found in many types of human cancers and is associated with chemoresistance, disease progression and poor prognosis including in PDAC [167]. Smac-mimetics may therefore restore and promote the induction of apoptosis through apoptotic signaling pathways in cancer cells and inactivate the NF-κB-mediated survival pathway [309]. Expression of Smac is also altered in many cancer types (reviewed [310]), including PDAC, where downregulation is mediated by heat-shock transcription factor 1 (HSF1) [311]. Therefore, a therapeutic approach that results in the degradation of cIAPs and/or antagonism of XIAP (X-linked inhibitor of apoptosis) could promote the lethal effects of TNF on cancer cells, while shutting down the survival pathway mediated by TNF driven NF-κB expression. Indeed, RNA-interference knockdown of XIAP sensitized PDAC cells for gamma-irradiation-induced apoptosis. Therefore, a basis exists for targeting XIAP using small molecules, as a novel approach to enhance sensitivity of PDAC [312,313].

We, and others showed that Smac-mimetics work by promoting auto-ubiquitylation and proteasomal degradation of cIAP1 and cIAP2 [306,307,314,315]. Particularly in the presence of a stimulus such as TNF, chemotherapy or TLR ligand this results in the formation of a RIPK1: caspase-8 complex, caspase-8 activation and the induction of tumor cell death [306,309,316,317]. We validated the second generation Smac-mimetic, birinipant, as a clinical candidate with a much improved safety and tolerability profile compared with the first generation compounds [318]. We have also shown that birinapant has demonstrated anti-leukemic efficacy [319]. The excellent safety profile of the birinapant and caspase-8 inhibitor, emricasan, combination in vivo, also warrants birinapant for clinical investigation not only in hematologic cancers [319,320] but other solid cancers [318], perhaps in synergy with immune checkpoint inhibitors or CAR T-cells [309,321,322,323,324]. Recent proteomic analysis characterized up-regulation of IAP family proteins (BIRC5/survivin, BIRC6/Bruce) and the E3 ubiquitin ligase class (Ring2, similar functions as IAP) as the mechanism of action of combinative gemcitabine and birinapant in human PDAC [325]. The major beneficial mechanism of this combinative treatment appeared to be down-regulation by birinapant of gemcitabine-mediated activation of DNA damage response regulation, DNA repair and strong activation of NF-κB. The combination of birinapant with gemcitabine also partially shifted NF-κB signaling toward a caspase-mediated pro-apoptotic signaling [325], confirming the IAP family as an important contributor to gemcitabine resistance and birinapant as a good drug target candidate for PDAC.

A number of other Smac-mimetic compounds have been developed (reviewed [309,310]). Recent studies also highlight the potential for Smac-mimetics specifically in PDAC. For instance, the Smac-mimetic JP1201 combined with gemcitabine decreased PDAC size and increased survival in pre-clinical murine models [326]. Smac-mimetics tested on PDAC cells as single agents such as SW IV-134 [327] or with other Smac-mimetics (AZD5582) [328] have demonstrated pro-apoptotic anti-tumor effects. Eight Smac-mimetics have been or are currently being evaluated in clinical trials for hematopoietic and solid cancers [310], including; Debio 1143/AT-406/SM-406, LCL161, APG1387, BI 891065 and ASTX660 [309,310], some of which are also currently being trialed for PDAC (Table 1). Since their cytotoxicity is often augmented when used in combination with other agents such as TNF, TRAIL or combined with gemcitabine [309,310,325,329], this approach is also being trialed for PDAC (Table 1).

While these studies are promising and nearly always seem to show a tumor specific killing or sensitization effect, a note of caution particularly in light of the discussion about the pro and anti-tumor roles of NF-κB is warranted. Since cIAPs inhibit non-canonical NF-κB it would most likely be counter-productive to use Smac-mimetics in tumors driven by non-canonical NF-κB. This principle has been demonstrated in a Myc driven lymphoma model. Since lymphoma growth is driven by non-canonical NF-κB it was not too surprising that the Smac-mimetic LCL-161 actually reduced survival in mice bearing these leukemias [330]. This highlights that the outcome of IAP inhibition is dependent on the dynamics of the NF-κB activities in any particular disease process and thus must assessed rigorously tested during pre-clinical studies [240,309,310].

Overall more research is required to determine how canonical and noncanonical NF-κB work to drive PDAC and if they can be effectively targeted. Due to the complexity of PDAC and its chemoresistance profile, combinative therapies targeting both NF-κB pathways in conjunction with conventional or targeted therapies could be effective, particularly if tailored to the molecular sub-type of the patient.

## 7. Conclusions

Overall, extensive studies of NF-κB and its relationship with PDAC have shown that both canonical and non-canonical NF-κB activation contribute to PDAC initiation, progression and metastasis. Further, the cross-talk of NF-κB with other signaling pathways indicates that it is the intracellular connections that are critical to development of this cancer. It is therefore an opportune time for the development of novel drugs that target the NF-κB signaling pathway in this context. Several agents have been discovered already and more are in the pipeline. Combination therapy of parts of the FOLFIRINOX regime with NF-κB inhibitors in combination with immune checkpoint blockade could be beneficial in order to treat PDAC or prevent chemotherapy resistance. Since NF-κB is expressed not only in PDAC but also the various cell types constituting the tumor inflammatory milieu, targeting of NF-κB, therefore should be directed at balancing overall NF-κB signaling within the whole tumor microenvironment. Extensive studies on animal models followed by clinical trials are required to fully understand the mode of action of these drugs in the tumor microenvironment.

## Figures and Tables

**Table 1:** In Progress Smac-Mimetic Clinical Trials for Pancreatic Cancer.

Smac-Mimetic	Adjuvant Therapy	Phase	Clinical Trial/Start Date	Status (12 February 2021)	Trial Centres
Debio 1143 ^a^	Pembrolizumab	I	NCT03871959/Sep-19	Recruiting	France
Debio 1143 ^b^	Nivolumab	I/II	NCT04122625/Apr-19	Recruiting	USA, France, Spain
LCL161	Gemcitabine/Nab-Paclitaxel	I	NCT01934634/Mar-14	Unknown	USA
APG1387 ^c^	None	I/II	NCT03386526/Nov-17	Recruiting	USA
APG1387 ^b^	None	Ib	NCT04284488/Dec-17	Recruiting	USA
APG1387	Gemcitabine/Nab-Paclitaxel	I/II	NCT04643405/Nov-20	Not yet recruiting	China
BI 891065 ^b^	Immunotherapy (BI 754091)	I	NCT04138823/Oct-19	Active, not recruiting	Japan
ASTX660 ^d^	None	I/II	NCT02503423/July-15	Recruiting	USA

^a^ Including advanced colorectal adenocarcinomas (colon/rectum). ^b^ Including other solid tumor types. ^c^ Including other advanced solid tumors or hematological malignancies. ^d^ Including other solid tumor types and lymphomas.

## Data Availability

Not applicable.
